# Supplementary material for: Classification of soybean seeds based on RGB reconstruction of hyperspectral images
Source: PLoS One. 2024 Sep 4;19(9):e0307329. doi: 10.1371/journal.pone.0307329 (PMC11373792; doi:10.1371/journal.pone.0307329)
Supplement: Attachment — Submitted filename: Response to Reviewers.docx [file pone.0307329.s001.docx]

Response to Reviewer1Comments

Dear reviewer:

Thank you for your decision and constructive comments on my manuscript. We have carefully considered the suggestion of Reviewer and make some changes. The blue part that has been revised according to your comments. Revision notes, point-to-point,aregiven as follows:

*Reviewer #1: This study discusses the development of a hyperspectral imaging approach for classifying soybean seeds that uses hyperspectral RGB picture reconstruction. To this end, they collected hyperspectral images of seven varieties of soybean through hyperspectral imager, and by employing the principle of the three base colors, the R, G and B bands were selected. This was complemented by reconstructing the images with different texture and color characteristics to generate a new dataset for seed segmentation. This was concluded by comparing the classification effect of the seven models. Experimental assessments supported the claim and the authors tried to prove the proposed approach with different set of analyses. While the work is comprehensive and supported by extensive data, there are some works in the literature with analogous methods that have not been discussed in the current manuscript, such as: Sensors 2019 Dec; 19(23): 5225, Front Plant Sci. 2022; 13: 1098864, and many other works can be found easily through a quick literature search. The authors should argue the novelty of the work compared to recent studies and show what parameters have been optimized and how the proposed technique addresses the current limitations compared to those studies.*

Response:  The original formulation was indeed a bit inappropriate, and has been changed and references have been added. The specific modification is: Lines 405 to 415 in the manuscript.

The addition reads: Feature band selection methods such as SPA in other literatures are based on spectral angles, and for full or hyperspectral data, a large amount of band information needs to be processed, and manually analyzing the spectral properties of each band can become very time-consuming and complex. The resulting bands for image reconstruction cannot improve the classification accuracy and other metrics of soybean seeds. The RGB reconstruction method proposed in this paper, which is based on the image perspective, uses the principle of three basic colors to change the color and texture characteristics of the image, and filters out some important bands before reconstruction, and the resulting new dataset has more obvious characteristics, and the classification accuracy of the nine datasets in the experiments and other metrics have been improved [28].

Here are some additional comments:

1) *The provided images need scalebars.*

Response: The actual diameter of a soybean is around 0.5cm, and the diameter of the soybean seed in the paper is around 1cm, magnified twice, with a scale of 0.5cm. Specific changes have been made to Figure 1 in the thesis by adding a scale label and changing the legend to a scale of 0.5 cm.

2) *What is the sample to result duration compared to other technologies?*

Response: You are right that time is also an important indicator that The specific modification is: Lines 362 to 368 in the manuscript.

The addition reads: The total test set times of ResNet18, ResNet34, ResNet50, ResNet101, CBAM-ResNet34, SENet-ResNet34, and SENet-ResNet34-DCN models are 3.41s, 4.05s, 3.37s, 3.75s, 2.95s, 2.83 s, 2.77s, and the SENet-ResNet34-DCN model has the shortest time, which is reduced by 0.06s-1.28s, and the test times for a single image are 0.0045s, 0.0053s, 0.0044s, 0.0049s, 0.0038s, 0.0037s, and 0.0036s, respectively. The SENet-ResNet34-DCN model reduced it by 0.0001s-0.0023s.

3) *The precision of the proposed system should be discussed further and evaluated quantitatively.*

Response: In the hyperspectral image RGB reconstruction into a new dataset part, only used the accuracy of an indicator as a reference is not very comprehensive, in the text I added precision, F1-score, recall, and other indicators for a comprehensive analysis. Figure 6 has also been modified. The accuracy is also analyzed in the subsequent algorithm.The specific modification is: Lines 222 to 230 and 361 to 362 of the manuscript.

The addition reads: The original dataset 188-120-60 has a classification accuracy of 88.87% and a precision of 89%, and the nine combinations have a 2% improvement in classification accuracy and precision.The 180-77-37 dataset has a classification accuracy of 90.18% and a precision of 90.43%, which is the least improvement.The 188-83-41 dataset has the highest classification accuracy of 91.75% and the highest precision of 91.71%, which is the most improvement. 91.71%, with the most improvement, 2.88% and 2.71% over the original dataset. Therefore the 188-83-41 dataset is referred to as the new dataset and applied to the following algorithm for soybean seed classification.( 222 to 230)

The  precision of SENet-ResNet34-DCN is 94.14%, which is 2.28% better than that of ResNet34.(361-362)

Thanks to the professional comments again that point out the above problems.The authors hope these explanations would answer your doubts.

Response to Reviewer2Comments

We thank the reviewer for the positive and constructive comments regarding our paper. The blue part that has been revised according to your comments. Revision notes, point-to-point,aregiven as follows:

*Reviewer #2: Overall, the quality of the paper is good. The dataset samples could be considered more for evaluation purposes. The analysis of the results could be improved. The extensive literature review has been made*.

Response:  The original formulation was indeed a bit inappropriate, and has been changed and references have been added. The specific modification is: Lines 405 to 415 in the manuscript.

The addition reads: Feature band selection methods such as SPA in other literatures are based on spectral angles, and for full or hyperspectral data, a large amount of band information needs to be processed, and manually analyzing the spectral properties of each band can become very time-consuming and complex. The resulting bands for image reconstruction cannot improve the classification accuracy and other metrics of soybean seeds. The RGB reconstruction method proposed in this paper, which is based on the image perspective, uses the principle of three basic colors to change the color and texture characteristics of the image, and filters out some important bands before reconstruction, and the resulting new dataset has more obvious characteristics, and the classification accuracy of the nine datasets in the experiments and other metrics have been improved [28].

Closing comment to Reviewer 2: We hope the revised manuscripts is now acceptable to you. If not, we are glad to receive any further feedback which we shall continue to apply our best effort to address.

During this dissertation revision process we identified several problems and addressed them.

(1) The order of authors was correct in the manuscript, but the second and third authors were reversed when submitting to the system, and will be corrected in this submission.
(2) Problem with figure, figure 12abcd was uploaded incorrectly, this submission will be corrected on the system.
(3) All the figures inside the paper have been adjusted by PACE, this time uploading the modified figures.
